# Physicians' experiences with end-of-life decision-making: Survey in 6 European countries and Australia

**DOI:** 10.1186/1741-7015-6-4

**Published:** 2008-02-12

**Authors:** Rurik Löfmark, Tore Nilstun, Colleen Cartwright, Susanne Fischer, Agnes van der Heide, Freddy Mortier, Michael Norup, Lorenzo Simonato, Bregje D Onwuteaka-Philipsen, the EURELD Consortium

**Affiliations:** 1 Centre for Bioethics at Karolinska Institutet and Uppsala Universitet, LIME, SE-171 77 Stockholm, Sweden; 2 Department of Medical Ethics, BMC C 13, Lund University, S-211 84 Lund, Sweden; 3 Southern Cross University, Hogbin Drive, Coffs Harbour NSW 2450 Australia; 4 Center for Health Sciences, School of Health Professions Zurich University of Applied Sciences, P.O. Box, CH-8401 Winterthur, Switzerland; 5 Department of Public Health, Erasmus MC, P.O. Box 2040, 3000 CA, Rotterdam, The Netherlands; 6 Ghent University, Ghent Bioethics Centre, Blandijnberg 2, 9000, Ghent, Belgium; 7 Unit of Medical Philosophy Department of Health Services Research, Institute of Public Health University of Copenhagen, Øster Farimagsgade 5, PO Box 2099, DK 1014 Denmark; 8 Department of Environmental Medicine and Public Health, University of Padova, Via Loredan 19, 35131 Padova, Italy; 9 Vrije Universiteit Medical Centre, Department of Public and Occupational Health and EMGO Institute, van der Boechorststraat 7, 1081 BT Amsterdam, the Netherlands

## Abstract

**Background:**

In this study we investigated (a) to what extent physicians have experience with performing a range of end-of-life decisions (ELDs), (b) if they have no experience with performing an ELD, would they be willing to do so under certain conditions and (c) which background characteristics are associated with having experience with/or being willing to make such ELDs.

**Methods:**

An anonymous questionnaire was sent to 16,486 physicians from specialities in which death is common: Australia, Belgium, Denmark, Italy, the Netherlands, Sweden and Switzerland.

**Results:**

The response rate differed between countries (39–68%). The experience of foregoing life-sustaining treatment ranged between 37% and 86%: intensifying the alleviation of pain or other symptoms while taking into account possible hastening of death between 57% and 95%, and experience with deep sedation until death between 12% and 46%. Receiving a request for hastening death differed between 34% and 71%, and intentionally hastening death on the explicit request of a patient between 1% and 56%.

**Conclusion:**

There are differences between countries in experiences with ELDs, in willingness to perform ELDs and in receiving requests for euthanasia or physician-assisted suicide. Foregoing treatment and intensifying alleviation of pain and symptoms are practiced and accepted by most physicians in all countries. Physicians with training in palliative care are more inclined to perform ELDs, as are those who attend to higher numbers of terminal patients. Thus, this seems not to be only a matter of opportunity, but also a matter of attitude.

## Background

Postponing death is not always a self-evident goal of medicine. Other goals have also to guide medical decision-making at the end of life, such as improving the quality of life of patients and their families through the prevention and relief of suffering, even if this might hasten death [1]. End-of-life decisions (ELDs) include decisions about withholding or withdrawing potentially life-prolonging treatment and about alleviation of pain or other symptoms with a possible life-shortening effect. In some countries it is also permissible to make decisions about euthanasia or physician-assisted suicide (EAS), defined as the administration, prescription or supply of drugs to end life at the patient's explicit request.

ELDs occur throughout the world, albeit at different rates for different actions [2-14]. In the first EURELD study, which presented data on 20,480 deaths in six European countries (Belgium, Denmark, Italy, the Netherlands, Sweden and Switzerland), physicians reported that death was preceded by an ELD in 23% (for Italy) to 51% (for Switzerland) of all deaths [2]. Most frequently it concerned alleviation of pain and symptoms (19–26%) and non-treatment decisions (4–28%). The treatment most frequently forgone was nutrition or hydration and medication (62–71%) [15]. Continuous deep sedation was provided in 2.5% of all deaths in Denmark and up to 8.5% in Italy. In 35% (Italy) to 64% (Denmark and The Netherlands) of deaths a decision was made to withhold or withdraw artificial nutrition or hydration [16]. EAS varied from 1% or less in Denmark, Italy, Sweden and Switzerland, to 1.82% in Belgium and 3.40% in the Netherlands [2].

A survey among physicians in Australia in 1996 used similar questions to the EURELD study described above, although using a different method. It was found that 31% of all Australian deaths were preceded by alleviation of pain and symptoms with possible life-shortening effect. Death was preceded by foregoing treatment in 29% of cases, and administration of drugs with the explicit intention of hastening death occurred in 5.3% (see [11]).

These studies looked at the frequency of ELDs in patient deaths. Few studies have investigated how much experience physicians have with performing ELDs. Such research could not only illuminate the willingness of physicians to perform ELDs, but also possible associations between physicians' background characteristics, attitudes and experience with ELDs. For the second EURELD study, Australia joined the consortium to participate in such a survey among physicians. The purpose of the present paper is to report (a) to what extent do physicians in the seven countries have experience with performing a range of ELDs, (b) if they have no experience with performing an ELD, would they be willing to do so under certain conditions and (c) which background characteristics are associated with having experience with/or being willing to make such ELDs. The specific questions are presented in Additional file 1.

## Methods

In each country, a random sample of 300 physicians was drawn from the professional registers of specialities in which physicians frequently attend to dying patients: these included anaesthesiology, general practice, geriatrics, gynaecology, internal medicine, neurology, nursing home medicine (the Netherlands only), oncology (not a separate speciality in the Netherlands), pulmonology and surgery. In Italy the sampling was drawn from hospital and general practice registers. When there were less than 300 physicians working in a speciality, all specialists were included in the sample. In Italy general practitioners were over-sampled. The number of questionnaires sent out in the end of 2002 varied from 1870 in Denmark to 3873 in Italy. In addition to background characteristics and palliative care education, physicians were asked about: their attitudes, intended behaviour and practices concerning end-of-life care; communication with terminally ill competent patients and their families; and experiences of making ELDs. The ELDs were described as neutrally and factually as possible in order to avoid differences in interpretation. EAS, for instance, were formulated as 'administering, prescribing or supplying drugs with the explicit intention of hastening the end of life on the explicit request of a patient'. The data of all countries were combined in a common database to ensure identical coding and analysis procedures. When presenting frequencies, weighting factors were used to correct for the different sampling fractions and response percentages in the different strata. Detailed inclusion criteria and methods have previously been reported [10].

For each country, the percentage of physicians who had (a) performed an ELD, (b) never performed an ELD, but would be willing to do so under certain conditions, (c) never performed an ELD and would never do so and (d) ever received an explicit request from a patient to administer, prescribe or supply drugs with the explicit intention of hastening death are presented.

Possible predictors for experiences with ELDs were analysed by multivariate and multinomial logistic regressions. Having performed an ELD versus never having performed an ELD (and willingness yes or no) was analysed using a multiple logistic regression per ELD entering sex (M, F), age (≤50, >50), palliative care training (yes, no), number of terminal patients in 12 months (≤5, >5), religion (religion (very) important, philosophy of life (very) important, religion and philosophy of life not important), attitude on right to decide to hasten death (strongly agree, agree, neutral, disagree, strongly disagree) and attitude on aiming at preserving life (strongly agree, agree, neutral, disagree, strongly disagree); these factors were controlled for country and clinical speciality. The same factors per ELD were used in order to make comparisons between ELDs possible. In addition, a multivariate logistic regression was performed in order to investigate the importance of clinical speciality on experiences with ELDs, adjusted for country. Finally, a multinomial logistic regression was used to compare the reference group of physicians with experience of EAS, with two groups of physicians who had never performed EAS: (1) physicians willing to perform EAS and (2) physicians unwilling to do so. Independent variables were: ever received an explicit request for physician-assisted death (PAD) (yes/no)

As no patients were involved in the study and that answering of the questionnaire was voluntary and anonymous, assessment by an ethics review committee was not required except in Australia where ethics approval was given by the Human Research Ethical Review Committee of the University of Queensland.

## Results

Response rates were: Australia (AU) 53% (*n *= 1478), Belgium (BE) 58% (*n *= 1750), Denmark (DE) 68% (*n *= 1217), Italy (IT) 39% (*n *= 1508), the Netherlands (NL) 61% (*n *= 1275), Sweden (SE) 60% (*n *= 1514) and Switzerland (CH) 64% (*n *= 1397). Altogether 10,139 questionnaires were studied.

Frequencies of experience with the different ELDs, and willingness to perform if not performed yet, are presented in Table 1. Physicians in Italy had the lowest experience of foregoing life-sustaining treatment (37% versus 72–86%) and the highest intention of not doing so (24% versus 1–6%). In all countries a majority of physicians had intensified the alleviation of pain or other symptoms while taking into account possible hastening of death. For Australia, Belgium, Denmark and the Netherlands this was a very large majority (83–95%), while the percentage was somewhat smaller in Italy (57%), Sweden (64%) and Switzerland (71%). The group of physicians who would never be willing to alleviate pain and other symptoms by increasing medication to a level that risked hastening death was largest in Italy (17% versus 1–10%). Experience with deep sedation until death was lowest in Italy (12%) and highest in the Netherlands (46%). Physicians in the Netherlands had most experience with receiving a request for hastening death (71%), followed by physicians from Belgium (40%), Switzerland (37%), Australia (36%) and Denmark (34%). Intentionally hastening death on the explicit request of a patient was most common in the Netherlands (56%). In the other countries the percentage of physicians who would never be willing to do this varied from 36% in Belgium to 84% in Sweden. In Italy this question was not asked.

**Table 1:** Experiences with end-of-life decisions (weighted rounded percentages). Missing observations per country: between 28 and 137 for withholding and withdrawing treatment; between 15 and 308 for intensifying treatment of pain and symptoms; between 0 and 64 for deep sedation; between 0 and 60 for receiving a request and between 65 and 330 for ending of life on request.

	**Au **(*n *= 1478)		**Be **(*n *= 1750)		**CH **(*n *= 1397)		**DK **(*n *= 1217)		**IT **(*n *= 1508)		**NL **(*n *= 1275)		**SE **(*n *= 1514)	
	
	%	95%CI	%	95%CI	%	95%CI	%	95%CI	%	95%CI	%	95%CI	%	95%CI
Withholding and/or withdrawing treatment taking into account the probability or certainty that this would hasten the end of the patient's life														
- ever	77	70–84	80	73–88	78	72–84	86	78–95	37	34–41	82	74–89	72	66–78
- never, but would be willing to do so under certain conditions	17	14–21	17	13–20	19	16–21	11	7–14	38	35–41	18	14–22	24	20–28
- never, and would never do so	6	4–8	3	1–4	4	2–5	4	1–6	24	22–27	1	0.2–1	5	3–6
Intensifying the alleviation of pain and/or symptoms by using drugs, taking into account the probability or certainty that this would hasten the end of the patient's life														
- ever	83	75–90	92	83–99	71	66–77	95	86–99	57	53–61	94	86–99	64	58–70
- never, but would be willing to do so under certain conditions	13	10–16	8	8–10	23	20–26	3	1–5	26	23–29	5	3–7	26	22–30
- never, and would never do so	4	2–6	1	0.1–2	5	5–8	2	1–4	17	15–19	1	0.1–1	10	8–13
Administering drugs to keep a patient in deep sedation until death, without giving (artificial) hydration or nutrition														
- ever	28	23–32	32	27–37	26	22–29	31	25–36	12	11–14	46	39–52	20	17–23
- never‡	72	65–80	68	61–75	74	69–80	70	62–77	88	83–93	55	48–61	80	74–87
Receiving an explicit request from a patient to administer, prescribe or supply drugs with the explicit intention of hastening the end of life														
- ever	36	31–41	40	34–45	37	33–41	34	28–39	14	12–16	71	63–78	24	20–27
-never	64	58–71	60	54–67	63	58–68	66	59–74	86	81–91	30	25–34	76	70–83
Administering, prescribing or supplying drugs with the explicit intention of hastening the end of life on the explicit request of a patient														
- ever	7	4–9	19	15–24	9	7–11	14	10–19	†		56	48–63	1	0.4–2
- never, but would be willing to do so under certain conditions	28	23–32	45	39–51	32	29–36	24	18–29	†		29	25–34	15	12–17
- never, and would never do so	66	59–73	36	30–41	59	53–64	62	54–70	†		15	12–18	84	78–91

† Question not asked in Italy because this would presumably upset the public opinion.

‡ Mistakenly, in the question on deep sedation no distinction was made between 'never, but would be willing...' and 'would never do so

Different factors were important for experiences with ELDs (Table 2). Female physicians less frequently had experience with all ELDs than male physicians. Physicians aged over 50 had less experience than younger physicians with withholding or withdrawing treatment and intensifying pain or symptom medication which risked hastening death, while they had more experience with the other ELDs. Both having had training in palliative care and having attended more than five terminal patients in the last 12 months resulted in more experience with all ELDs. Religion being important for one's professional attitude was associated negatively with intensifying pain or symptom medication and hastening death on a patient's request. A non-religious philosophy being important for one's professional attitudes had the opposite effect. Being of the opinion that every person has a right to decide to hasten death was positively associated with experience with all ELDs except withholding or withdrawing treatment. Being of the opinion that physicians should always aim at preserving life was negatively associated with experience with all ELDs and most strongly with intentionally hastening death (odds ratio (OR) = 0.44) and withholding or withdrawing treatment (OR = 0.55).

**Table 2:** Importance of various factors on experience with ELDs (*n *= 6587, ORs). Multivariate logistic regression; adjusted for country and clinical specialty.

	Female	>50 years	training in palliative care	>5 terminal patients in 12 months	Religion (very) important	Philosophy of life (very) important	Right to decide to hasten the end of life	Physicians should aim at preserving life
Ever withheld or withdrawn treatment	0.70*	0.88*	1.7*	2.7*	0.94	1.2	1.1	0.55*
Ever intensified alleviation of pain and symptoms	0.75*	0.85*	1.8*	2.3*	0.83*	1.3*	1.2*	0.63*
Ever deeply sedated a patient until death	0.78*	1.2*	1.5*	1.8*	0.96	1.1	1.3*	0.70*
Ever received a request for ending of life	0.71*	1.2*	1.7*	1.9*	1.0	1.3*	1.2*	0.73*
Ever ended the life of a patient on his or her request	0.63*	1.6*	1.3*	1.4*	0.82*	1.6*	2.9*	0.44*

* OR differs significantly from 1.0 (α = 0.05).

Compared with general practitioners, gynaecologists, neurologists and surgeons seemed to have the lowest experience of most ELDs. Geriatricians (OR = 3.1), physicians in internal medicine (OR = 2.0), anaesthesiologists (OR = 1.9) and oncologists (OR = 1.4) had more experience with foregoing treatment than general practitioners. Oncologists and pulmonologists had more experience than general practitioners with all ELDs except hastening death on the explicit request of a patient. (Table 3).

**Table 3:** Importance of clinical specialty on experience with end-of-life care (*n *= 6587, ORs). Multivariate logistic regression (reference group: general practitioners); adjusted for country.

	Anaesthesiology	Geriatrics	Gynaecology	Internal medicine	Neurology	Oncology	Pulmonology	Surgery
Ever withheld or withdrawn treatment	1.9*	3.1*	0.55*	2.0*	1.2	1.4*	2.0	1.5
Ever intensified alleviation of pain and symptoms	1.2*	1.2	0.55*	1.7*	0.74*	1.8*	2.2*	1.2
Ever deeply sedated a patient until death	1.0	1.0	0.51*	0.98	0.72*	2.4*	1.3*	0.76*
Ever received a request for ending of life	0.57*	1.4*	0.42*	0.96	0.87	1.9*	1.5*	0.80*
Ever ended the life of a patient on his or her request	0.50*	0.34*	0.37*	0.51*	0.51*	0.90	0.81	0.46*

* Odds ratio differs significantly from 1.0 (α = 0.05).

Table 4 shows the results of a multinomial logistic regression aimed at identifying factors associated with (n)ever having hastened death among three groups: physicians who have performed this ELD, physicians who never did but would be willing to do so under certain conditions and physicians who never did and would never do so. Compared with physicians who had hastened death, physicians who would never do so were less likely to be over 50 years of age, to have had palliative care training, to have attended to more than five terminal patients in the last year, to have a non-religious philosophy of life and were also less likely to think that a person should have the right to decide whether or not to hasten their death; they were more likely to be female, to think that physicians always should aim at preserving the lives of their patient, that sufficient availability of high-quality palliative care prevents almost all requests for euthanasia, that permitting hastening death on the explicit request of a patient will lead to hastening death without a request and will harm the relationship between patients and physicians. Looking at the physicians who never hastened death on the request of a patient but would be willing to do so under certain conditions shows a similar pattern, albeit that the associations are less strong.

**Table 4:** Comparison of importance of various factors for never performed EAS, but being willing to do so under certain conditions and never performed EAS, and not willing to ever do so (*n *= 6348, ORs). Multinomial logistic regression; reference group 'ever performed EAS'; adjusted for country and clinical specialty.

	**Never performed EAS, but willing to do so under certain conditions**	**Never performed EAS, and would never do so**
Being female	1.5*	1.8*
Being over 50 years	0.64*	0.55*
Ever having had palliative care training	0.77*	0.72*
Attending to more than 5 terminal patients in 12 months	0.65*	0.73*
Religion being (very) important for professional attitudes	0.95	1.2
Non-religious philosophy of life being (very) important for professional attitudes	0.78	0.45*
(Strongly) agreeing with the statement 'a person should have the right to decide whether or not to hasten the end of his or her life'	0.72*	0.23*
(Strongly) agreeing with the statement 'In all circumstances physicians should aim at preserving the lives of their patients, even if patients ask for the hastening of the end of their lives'	1.0	2.5*
(Strongly) agreeing with the statement 'sufficient availability of high-quality palliative care prevents almost all requests for euthanasia or assisted suicide'	1.4*	2.1*
(Strongly) agreeing with the statement 'permitting the use of drugs in lethal doses on the explicit request of the patient will gradually lead to an increase in the use of drugs in lethal doses without a request of the patient'	1.0	1.5*
(Strongly) agreeing with the statement 'permitting the use of drugs in lethal doses on the explicit request of the patient will harm the relationship between patients and physicians'	1.1	2.5*

* Odds ratio differs significantly from 1.0 (α = 0.05).

## Discussion

There are differences between physicians in the countries under study regarding experiences with ELDs, willingness to perform ELDs and frequency with which requests for EAS are received. In general, physicians in Italy have least experience with these issues, followed at some distance by Sweden, while physicians in the Netherlands have the most experience. Foregoing treatment and alleviation of pain and symptoms by intensifying medication to a level, which risks hastening death are accepted by physicians in all countries, since only a small minority have never performed them and would never do so. These are also the ELDs that were found to occur most frequently in the first EURELD (death certificate) study.

The main strength of this study was that the same methodology was used in all countries, which made it possible to compare countries. We are not aware of any other international surveys on this subject. A limitation of this study is that it was retrospective, which may have resulted in recall bias.

A number of the authors currently work or research in end-of-life areas. Our experience suggests that since physicians sent in this questionnaire in 2003, the question arises whether there are reasons to believe that physicians' practices have changed since then. In the Netherlands, ELDs have been studied extensively since then [17]. It showed that the practice of ELDs remained stable over the years, with the exception of euthanasia, which occurred less frequently in 2005 compared with 2001. In Belgium things may have changed, as the euthanasia law is only now in full effect. Recent data obtained by another method than the death certificate method suggest that there is more euthanasia in Flanders than before, 1.6% in 2005–2006 instead of 0.3%. There is also a rise in the total number of potentially life-shortening ELDs, from 38% to 50% [18]. The general increase may of course be reflected in physicians' experiences with ELDs.

Although reasons for the differences between countries can only be speculative, in the Netherlands the reason that physicians have more experience with ELDs may be a more liberal tradition and higher respect for patient autonomy. A religious influence is not evident, as Belgium, with a substantial Catholic population, has the second highest experience with ELD, and Sweden, which is a Protestant country, has the lowest together with Italy, a Catholic country. Denmark, which also has a Protestant population, is closest to Belgium. The results do indicate that a non-religious philosophy of life seems to increase the willingness to perform EAS, possibly out of respect for patient autonomy. Cohen et al [19] have studied life-stance and general attitudes towards ELDs in more detail. They found that teachings of religious bodies indeed have an influence on end-of-life decision-making, but are certainly not blankly accepted by physicians. The influence of doctrinal teachings is somewhat clearer on general attitudes towards end-of-life decision-making. It can perhaps be explained by the fact that most people embrace (theistic) belief not in strict metaphysical terms, but in non-imperative ways, allowing for adaptation to particular situations, for instance to the needs and wishes of the dying and to considerations of humaneness.

Physicians can only perform EAS when a patient requests it. Physicians in all countries receive euthanasia requests, most often in the Netherlands, where physicians also have most experience with performing EAS.

The results show that physicians with training in palliative care are more inclined to make ELDs. While this may be expected for some of the ELDs, it is somewhat surprising for EAS. One hypothesis may be that palliative care physicians develop a higher attention to patients' wishes. Further research is needed to clarify and explain this finding.

Furthermore, the findings indicate that the legislation and medical guidelines are reflected in physicians' experiences. In all countries, physicians had the highest experiences of non-treatment decisions and alleviation of pain and other symptoms with possible life-shortening effect: kinds of ELDs, which are legal in all participating countries. The fact that experiences of continuous deep sedation, which is legal in all countries, is relatively low, demonstrates that this ELD is more strongly influenced by situational factors such as uncontrollable pain and symptoms than by legal regulations. The different legal regulations concerning EAS are also reflected in physicians' experiences. Shortly before this study was performed, the Netherlands and Belgium changed their legislation, in 2001 and 2002, and now permit EAS under certain conditions. In the Netherlands EAS are regulated as two possible end-of-life options. In Belgium the law only regulates euthanasia. In both countries the patient involved must be a mentally competent adult when requesting help. Doctors can only proceed when they know the patient well enough to be able to assess whether their request for euthanasia is voluntary and well-considered, whether the patients' medical situation is without prospect of improvement and whether the individual's suffering is unbearable. The ability to refuse a request for euthanasia guarantees a doctor's freedom of conscience in both countries [20]. Whether this has influenced experiences and attitudes remains to be studied in Belgium. For the Netherlands, the evaluation of the euthanasia law showed that the incidence of EAS decreased from 2.8% in 2001 to 1.8% in 2005 (see [17]).

In Switzerland, assistance in suicide is allowed provided that the person seeking assistance has decisional capacity and the person assisting is not motivated by reasons of self-interest; euthanasia is forbidden in all circumstances. Experiences with ELDs can be associated with two types of factors. One is the opportunity the physician has for making ELDs. The second is the attitude of the physician towards questions about philosophy of life, e.g. whether people have a right to decide to hasten the end of life and whether physicians should always aim at preserving life. Older physicians may have been practising medicine longer and thereby have an increased chance of ever having performed an ELD. Further, the number of terminal patients attended to by the physician within a given time period varies from one speciality to another. However, since having had palliative care training is positively associated with having experience with all ELDs, independent of the number of terminal patients under the physician's care, this factor probably reflects an attitude. Female physicians have less experience with ELDs, which does not seem to be related to opportunity and attitude. A similar finding comes from Italy, where male anaesthesiologists had greater experience with foregoing treatment [21]. However, the reason is not obvious and ought to be studied in the future.

## Conclusion

In conclusion, there are differences between countries in experiences with ELDs, in willingness to perform ELDs and in receiving requests for EAS. Foregoing treatment and intensifying alleviation of pain and symptoms are practiced and accepted by most physicians in all countries. Physicians with training in palliative care are more inclined to perform ELDs, as are those who attend to higher numbers of terminal patients. Thus, this seems not to be only a matter of opportunity, but also a matter of attitude.

## Competing interests

The author(s) declare that they have no competing interests.

## Authors' contributions

RL and TN drafted the manuscript. BDOP performed the statistical analyses and drafted the manuscript. All authors contributed to the data collection together with the members of the EURELD consortium. All authors have contributed to the manuscript and approved the final version.

## Appendix

See Additional file 1

## Pre-publication history

The pre-publication history for this paper can be accessed here:



## Supplementary Material

Additional file 1 Part of the questionnaire: EURELD 2002 – questions regarding experiences and statements Click here for file

## References

[B1] Sepúlveda C, Marlin A, Yoshida T, Ullrich A (2002). Palliative care. The World Health Organisation's global perspective. J Pain Symptom Manage.

[B2] van der Heide A, Deliens L, Faisst K, Nilstun T, Norup M, Paci E, van der Wal G, van der Maas PJ, the EURELD consortium (2003). End-of-life decision-making in six European countries: descriptive study. Lancet.

[B3] Bosshard G, Fischer S, van der Heide A, Miccinesi G, Faisst K (2006). Intentionally hastening death by withholding or withdrawing treatment. Wien Klin Wochenschr.

[B4] van Delden JJ, Löfmark R, Deliens L, Bosshard G, Norup M, Cecioni R, van der Heide A, the EURELD consortium (2006). Do-not-resuscitate decisions in six European countries. Crit Care Med.

[B5] Buiting HM, van Delden JJM, Rietjens JAC, Onwuteaka-Philipsen B, Bilsen J, Fischer S, Löfmark R, Miccinesi G, Norup M, van der Heide A, the EURELD-Consortium (2007). Forgoing artificial nutrition or hydration in patients nearing death in six European countries. J Pain Symptom Manage.

[B6] Bilsen J, Norup M, Deliens L, Miccinesi G, van der Wal G, Löfmark R, Faisst K, van der Heide A, the EURELD Consortium (2006). Drugs used to alleviate symptoms with life shortening as a possible side effect: end-of-life care in six European countries. J Pain Symptom Manage.

[B7] Sprung CL, Cohen SL, Sjökvist P, Baras M, Bulow HH, Hovilehto S, Ledoux D, Lippert A, Maia P, Phelan D, Schobersberger W, Wennberg E, Woodcock T, Ethicus Study Group (2003). End-of-life practices in European intensive care units: the Ethicus Study. JAMA.

[B8] Førde R, Aasland OG, Falkum E (1997). The ethics of euthanasia – attitudes and practice among Norwegian physicians. Soc Sci Med.

[B9] Faber-Langendoen K (1996). A multi-institutional study of care given to patients dying in hospitals. Ethical and practice implications. Arch Intern Med.

[B10] Onwuteaka-Philipsen BD, Fisher S, Cartwright C, Deliens L, Miccinesi G, Norup M, Nilstun T, van der Heide A, van der Wal G, the European End-of-Life Consortium (2006). End-of-life decision making in Europe and Australia: a physician survey. Arch Intern Med.

[B11] Kuhse H, Singer P, Baume P, Clark M, Rickard M (1997). End-of-life decisions in Australian medical practice. Med J Aust.

[B12] Mitchell K, Owens G (2004). End-of-life decision-making by New Zealand general practitioners: a national survey. N Z Med J.

[B13] Seale C (2006). National survey of end-of-life decisions made by UK medical practitioners. Palliat Med.

[B14] White DB, Curtis JR, Lo B, Luce JM (2006). Decisions to limit life-sustaining treatment for critically ill patients who lack both decision-making capacity and surrogate decision-makers. Crit Care Med.

[B15] Bosshard G, Nilstun T, Bilsen J, Norup M, Miccinesi G, van Delden JJ, Faisst K, van der Heide A, the European End-of-Life Consortium (2005). Forgoing treatment at the end of life in 6 European countries. Arch Intern Med.

[B16] Miccinesi G, Rietjens JA, Deliens L, Paci E, Bosshard G, Nilstun T, Norup M, van der Wal G, the EURELD Consortium (2006). Continuous deep sedation: physicians' experiences in six European countries. J Pain Symptom Manage.

[B17] van der Heide A, Onwuteaka-Philipsen BD, Rurup ML, Buiting HM, van Delden JJ, Hanssen-de Wolf JE, Janssen AG, Pasman HR, Rietjens JA, Prins CJ, Deerenberg IM, Gevers JK, van der Maas PJ, van der Wal G (2007). End-of-life practices in the Netherlands under the Euthanasia Act. N Engl J Med.

[B18] Van den Block L (2007). Het sterfbed in België Resultaten van de SENTI-MELC studie 2005–2006.

[B19] Cohen J, van Delden J, Mortier F, Löfmark R, Norup M, Cartwright C, Faisst K, Canova C, Onwuteaka-Philipsen B, Bilsen J, on behalf of the EURELD Consortium The influence of physicians' life-stance on attitudes towards end-of-life decisions and actual end-of-life decision-making in six countries. J Med Ethics.

[B20] Deliens L, van der Wal G (2003). The euthanasia law in Belgium and the Netherlands. Lancet.

[B21] Giannini A, Pessina A, Tacchi EM (2003). End-of-life decisions in intensive care units: attitudes of pshysicians in an Italian urban setting. Intensive Care Med.

