# Supplementary material for: Physicians' experiences with end-of-life decision-making: Survey in 6 European countries and Australia
Source: BMC Med. 2008 Feb 12;6:4. doi: 10.1186/1741-7015-6-4 (PMC2277432; doi:10.1186/1741-7015-6-4)
Supplement: Additional file 1 — Part of the questionnaire: EURELD 2002 – questions regarding experiences and statements [file 1741-7015-6-4-S1.doc]

# **A. Specialty and work setting**

| **A1** | Are you currently working as a practising physician? |  | - Yes |
| --- | --- | --- | --- |
|  |  |  | - No 🡪 *end of questionnaire, please send the questionnaire back in enclosed reply envelope* |
|  |  |  |  |
| **A2** | What is the clinical specialty you are mainly working in at this moment? And for how many years have you been working in this specialty? Give only one answer |  | - Anaesthesiology………….……………….yearss |
|  |  |  | - General practice…..…….……………….years |
|  |  |  | - Geriatrics…….………….………………years |
|  |  |  | - Gynaecology…………….………………years |
|  |  |  | - Internal medicine……….……………….years |
|  |  |  | - Neurology..………………………………years |
|  |  |  | - Nursing home medicine...……………….years |
|  |  |  | - Oncology……….………………………..years |
|  |  |  | - Pulmonology………….…………………years |
|  |  |  | - Surgery………..….…………...…………years |
|  |  |  | - Other, namely ……………………………….   ………………………………………………..years |
|  |  |  |  |
| **A3** | In what kind of practice or institution are you working at this moment? And for how many years?  *More than one answer possible* |  | - General practice………………………….years - Home for the elderly..…………………...years - Hospital……………………………...…..years - Nursing home…………………...……….years - Other……………………………………..years |

**C. Statements**

Please indicate to what extent you agree or disagree with the following statements.

|  |  | strongly agree | agree | neutral | disagree | strongly disagree |
| --- | --- | --- | --- | --- | --- | --- |
|  |  |  |  |  |  |  |
| **C5** | A person should have the right to decide whether or not to hasten the end of his or her life |  |  |  |  |  |
|  |  |  |  |  |  |  |
| **C6** | Sufficient availability of high-quality palliative care prevents almost all requests for euthanasia or assisted suicide |  |  |  |  |  |
|  |  |  |  |  |  |  |
| **C9** | In all circumstances physicians should aim at preserving the lives of their patients, even if patients ask for the hastening of the end of their lives |  |  |  |  |  |
|  |  |  |  |  |  |  |
| **C10** | Permitting the use of drugs in lethal doses on the explicit request of the patient will gradually lead to an increase in the use of drugs in lethal doses without a request of the patient |  |  |  |  |  |
|  |  |  |  |  |  |  |
| **C13** | Permitting the use of drugs in lethal doses on the explicit request of the patient will harm the relationship between patients and physicians |  |  |  |  |  |
|  |  |  |  |  |  |  |

**D. Experience with end of life care**

| **D1** | Have you had any specific formal training in palliative care? If yes, please estimate for how many days (including contact hours and self study).  *more than one answer possible* |  | - No - Yes, in undergraduate medical study; …...days - Yes, in postgraduate medical study; ….….days - Yes, in postgraduate course, ……..………days - Yes, other, namely……………………….……   ……………………………………for ………..days |
| --- | --- | --- | --- |
| **D2** | Do you think formal training in palliative care should be extended?  *more than one answer possible* |  | - No - Yes, in undergraduate medical education - Yes, in postgraduate medical education - Yes, in postgraduate courses |
|  |  |  |  |
| **D3** | How many terminal patients were under your care during the last 12 months? |  |  |

| **D15** | Did you ever withhold or withdraw a treatment taking into account the probability or certainty that this would hasten the end of the patient’s life? | | |
| --- | --- | --- | --- |
|  | - Yes, withholding => When was the last time? months/years ago - Yes, withdrawing => When was the last time? months/years ago - No 🡪 Could you think of a situation in which you would do so? - Yes, only for withholding - Yes, only for withdrawing - Yes, both for withholding & withdrawing - No | | |
|  |  | | |
| **D16** | Did you ever intensify the alleviation of pain and/or symptoms by using drugs such as opioids, taking into account the probability or certainty that this would hasten the end of the patient’s life? | | |
|  | - Yes 🡪 When was the last time? months/years ago - No 🡪 Could you think of a situation in which you would do so? - Yes - No | | |
|  |  |  |  |
| **D17** | Did you ever administer drugs, such as benzodiazepines or barbiturates, to keep a patient in deep sedation until death, without giving (artificial) hydration or nutrition? | | |
|  | - Yes 🡪 When was the last time? months/years ago - No | | |
|  |  |  |  |
| **D18** | Did you ever receive an explicit request from a patient to administer, prescribe or supply drugs with the explicit intention of hastening the end of life? | | |
|  | - Yes 🡪 When was the last time? months/years ago      - No | | |
|  |  |  |  |
| **D19** | Did you ever on the explicit request of a patient administer, prescribe or supply drugs with the explicit intention of hastening the end of life? | | |
|  | - Yes, prescribed/supplied drugs => When was the last time? months/years ago - Yes, administered drugs => When was the last time? months/years ago - No 🡪 Could you think of a situation in which you would do so? - Yes, only for prescribing/supplying drugs - Yes, only for administering drugs - Yes, both for prescribing/supplying and administering drugs - No | | |

# E. Demographic characteristics

| **E1** | What is your sex? |  | - Male |
| --- | --- | --- | --- |
|  |  |  | - Female |
|  |  |  |  |
| **E2** | What is your age? |  | - <40 years |
|  |  |  | - 40-50 years |
|  |  |  | - >50 years |
|  |  |  |  |
| E3 | What do you consider to be your religion or philosophy of life? |  | - roman catholic |
|  |  |  | - protestant |
|  |  |  | - other religion, namely ……………………………. |
|  |  |  | - non-religious, namely ………………………… |
|  |  |  | - no specific philosophy |
|  |  |  |  |
| **E4** | How important is your religion or philosophy of life in your professional attitude towards end-of-life decision-making? |  | - very important |
|  |  |  | - important |
|  |  |  | - less important |
|  |  |  | - not important at all |

| Please provide here any comments on answers to the previous questions you wish to clarify or expand |
| --- |

**End of questionnaire, thank you for your co-operation!**

| *Please post the questionnaire in the enclosed prepaid envelope. Send back the reply card separately in order to avoid unnecessary reminders. Stamps are not needed.* |
| --- |
